# Synapse and Receptor Alterations in Two Different S100B-Induced Glaucoma-Like Models

**DOI:** 10.3390/ijms21196998

**Published:** 2020-09-23

**Authors:** Lara Benning, Sabrina Reinehr, Pia Grotegut, Sandra Kuehn, Gesa Stute, H. Burkhard Dick, Stephanie C. Joachim

**Affiliations:** Experimental Eye Research Institute, University Eye Hospital, Ruhr-University Bochum, In der Schornau 23-25, 44892 Bochum, Germany; Lara.Benning@uni-wh.de (L.B.); sabrina.reinehr@rub.de (S.R.); pia.grotegut@rub.de (P.G.); sandra.kuehn@rub.de (S.K.); gesa.stute@rub.de (G.S.); Burkhard.Dick@kk-bochum.de (H.B.D.)

**Keywords:** glaucoma, animal model, systemic immunization, intraocular injection, S100B protein, synapses, NMDA receptor, GABA receptor

## Abstract

Glaucoma is identified by an irreversible retinal ganglion cell (RGC) loss and optic nerve damage. Over the past few years, the immune system gained importance in its genesis. In a glaucoma-like animal model with intraocular S100B injection, RGC death occurs at 14 days. In an experimental autoimmune glaucoma model with systemic S100B immunization, a loss of RGCs is accompanied by a decreased synaptic signal at 28 days. Here, we aimed to study synaptic alterations in these two models. In one group, rats received a systemic S100B immunization (*n* = 7/group), while in the other group, S100B was injected intraocularly (*n* = 6–7/group). Both groups were compared to appropriate controls and investigated after 14 days. While inhibitory post-synapses remained unchanged in both models, excitatory post-synapses degenerated in animals with intraocular S100B injection (*p* = 0.03). Excitatory pre-synapses tendentially increased in animals with systemic S100B immunization (*p* = 0.08) and significantly decreased in intraocular ones (*p* = 0.04). Significantly more *N*-methyl-d-aspartate (NMDA) receptors (both *p* ≤ 0.04) as well as gamma-aminobutyric acid (GABA) receptors (both *p* < 0.03) were observed in S100B animals in both models. We assume that an upregulation of these receptors causes the interacting synapse types to degenerate. Heightened levels of excitatory pre-synapses could be explained by remodeling followed by degeneration.

## 1. Introduction

Glaucoma is the second leading cause for blindness, even before diabetic retinopathy, in Germany [1]. It is also one of the main reasons for blindness globally, together with age-related macular degeneration, cataract, or uncorrected refractive errors [2,3]. Whereas cataract and refractive errors are curable diseases, glaucoma leads to irreversible vision loss. According to estimates, in 2040, glaucoma will cause blindness or scotoma to nearly 111.8 million people [4], with an upward trend due to aging population [5,6]. As a neurodegenerative disease, glaucoma is characterized by morphological changes at the optic nerve head and retinal nerve fiber layer [7]. This leads to a loss of retinal ganglion cells (RGCs), resulting in visual field deficits [8].

Although the main risk factor is increased intraocular pressure (IOP), about 30% of glaucoma patients sicken independently from this; they suffer from so-called normal-tension glaucoma [9]. To this day, the exact pathomechanism of glaucoma remains unknown, thus it is inadequately treatable.

In previous clinical studies, glaucoma patients showed an increased antibody titer against the protein S100B in their tear film [10]. S100B is a calcium-binding protein, largely expressed by astrocytes [11]. It is well known that a high S100B level leads to increased microglia activation, resulting in a massive release of pro-inflammatory cytokines [12]. This inflammation can cause cell damage and is suggested to play a key role in neurodegenerative diseases, such as Alzheimer’s disease or multiple sclerosis, in which an increased S100B concentration is encountered as well [13,14]. This raises the question if glaucomatous damage is caused directly by inflammatory response or if there are more subsequent pathophysiological processes.

To determine the exact pathomechanism of glaucoma more precisely, different animal models have been established over the past few years. Since glaucoma is a multifactorial disease and the immune system has a reputation for playing a decisive role in its genesis [15,16,17,18,19,20,21,22,23,24], some of these animal models allow the IOP-independent investigation of pathomechanisms related to immunological changes [25,26]. In the experimental autoimmune glaucoma model (EAG), systemic S100B immunization leads to a significant loss of RGCs in rats after 28 days [27]. In an intraocular glaucoma-like model in rats, the injection of S100B results in significant RGC and amacrine cell death after 14 days and triggers a microglia response [28,29].

Besides these investigations in both models, possible changes in synaptic circuits have so far received little attention. EAG rats that received a systemic immunization with S100B in combination with heat shock protein 27 (HSP27) showed a significant loss of RGCs, bipolar cells, and amacrine cells, as well as a lower synapse signal after 28 days. Besides less post-synapses visualized by the post-synaptic density protein 95 (PSD-95), there were also fewer pre-synapses visualized by the bassoon protein [30]. This suggests that synaptic alterations could be involved in the pathomechanism of the glaucoma damage in these animal models.

Following this, the aim of the current study was to investigate the correlation between synaptic alterations and glaucoma pathomechanisms more precisely. For this purpose, rats were injected with the S100B protein systemically or intraocularly. Histological analyses were carried out after 14 days. Therefore, we were able to detect possible synapse changes prior to and simultaneously with RGC death. Inhibitory post-synapses, excitatory post-synapses, and excitatory pre-synapses were analyzed in retinas from both models. Since it is well known that PSD-95 interacts with the *N*-methyl-d-aspartate (NMDA) receptor 1 [31,32,33] and gephyrin with the gamma-aminobutyric acid (GABA) A receptor α3 [34], this could possibly affect synaptic plasticity.

## 2. Results

### 2.1. Inhibitory Post-Synapses Are Not Affected

**Figure 1:**
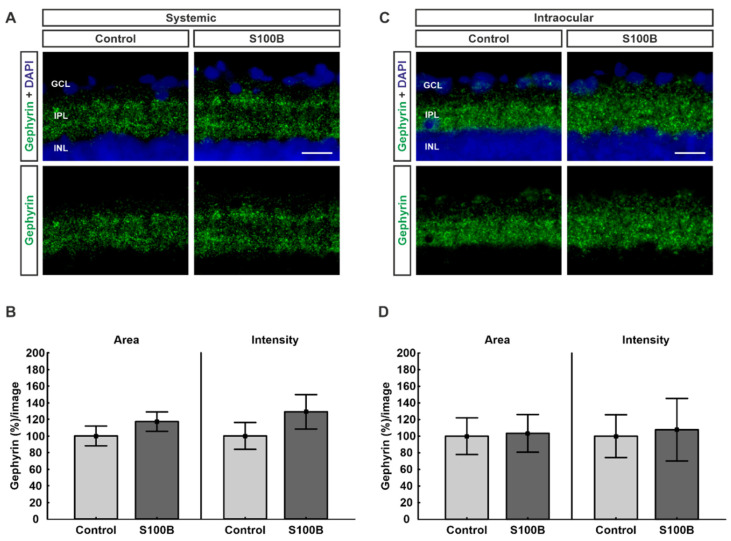
Gephyrin remains unchanged. (**A**) Retinas from systemically immunized animals (*n* = 7/group) were stained against gephyrin (green). Cell nuclei were visualized with 4′,6 diamidino-2-phenylindole (DAPI; blue). (**B**) In comparison to controls, no alterations could be observed in the gephyrin stained area (*p* = 0.31) and intensity (*p* = 0.29) in the S100B group. (**C**) Retinas from animals with intraocular S100B injection (*n* = 7/group) were stained against gephyrin (green). Cell nuclei were visualized with DAPI (blue). (**D**) No differences were noted regarding the percentage of stained area (*p* = 0.91) as well as the percentage of intensity (*p* = 0.86) between both groups. Abbreviations: GCL: ganglion cell layer; IPL: inner plexiform layer; INL: inner nuclear layer. Values are mean ± SEM. Scale bars: 20 µm.

To analyze inhibitory post-synapses, retinas were stained against gephyrin (Figure 1A,C). In systemically immunized S100B animals, the stained area remained unchanged (117.25 ± 11.6%; *p* = 0.31) in comparison to controls (100.00 ± 11.8%; Figure 1B). Furthermore, the intensity of the staining in these animals was not affected (129.01 ± 20.7%; *p* = 0.29) compared to controls (100.00 ± 16.1%; Figure 1B). Regarding the intraocular-injected animals, the percentage of the labeled area showed no differences between the S100B (103.38 ± 22.6%; *p* = 0.91) and the control group (100.00 ± 22.0%; Figure 1D). In addition, the intensity of the staining was unaltered (107.79 ± 37.6%; *p* = 0.86) in comparison to controls (100.00 ± 25.7%; Figure 1D).

### 2.2. Upregulation of GABA Receptors in Both Models

**Figure 2:**
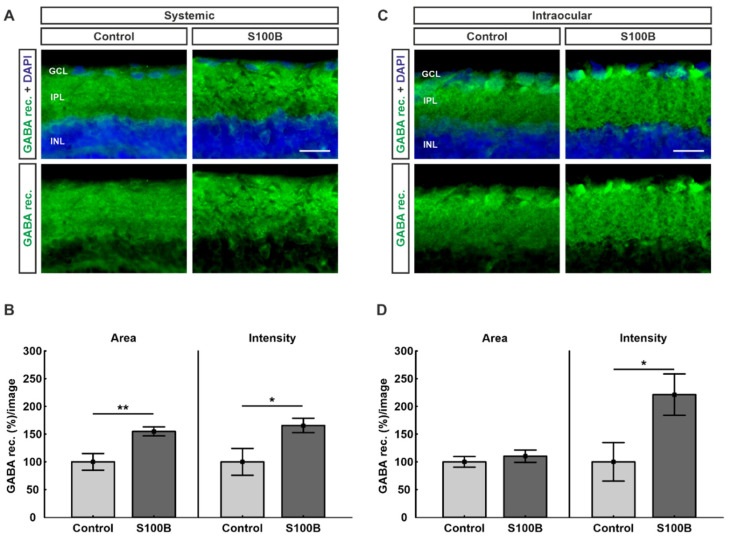
Upregulation of GABA receptors. (**A**) Retinas from animals immunized with S100B (*n* = 7/group) were labeled with antibodies against GABA receptors (green). Cell nuclei were stained with DAPI (blue). (**B**) In comparison to controls, the percentages of GABA receptor area (*p* = 0.007) and intensity (*p* = 0.03) were significantly increased in animals with systemic S100B immunization. (**C**) Retinas from intraocular-injected animals (*n* = 7/group) were labeled against GABA receptors (green). DAPI visualized cell nuclei (blue). (**D**) Statistical analyses showed that the intensity of the staining was significantly increased in the intraocular S100B group compared to controls (*p* = 0.03). The percentage of labeled area remained unchanged (*p* = 0.49). Abbreviations: GCL: ganglion cell layer; IPL: inner plexiform layer; INL: inner nuclear layer; GABA rec.: GABA receptor. Values are mean ± SEM. Scale bars: 20 µm. * *p* < 0.05, ** *p* < 0.01.

Due to the known interaction between gephyrin and GABA-A receptor α3, retinas were labeled with an antibody against GABA-A receptor α3 (Figure 2A,C). Animals that received a systemic S100B immunization showed a significant increase in their GABA-A receptor α3-labeled area (154.93 ± 8.1%; *p* = 0.007) compared to the control group (100.00 ± 15.0%; Figure 2B). Additionally, the intensity of the staining was significantly increased in the S100B group (165.64 ± 12.9%; *p* = 0.03) compared to controls (100.00 ± 24.2%; Figure 2B).

While the intraocular injection had no effect on the percentage of area between the S100B (110.21 ± 11.0%; *p* = 0.49) and the control group (100.00 ± 9.5%), the intensity of the staining was significantly increased in the S100B group (221.31 ± 37.3%; *p* = 0.03) in comparison to controls (100.00 ± 34.6%; Figure 2D).

### 2.3. Excitatory Post-Synapses Degenerated in Intraocular-Injected Animals

**Figure 3:**
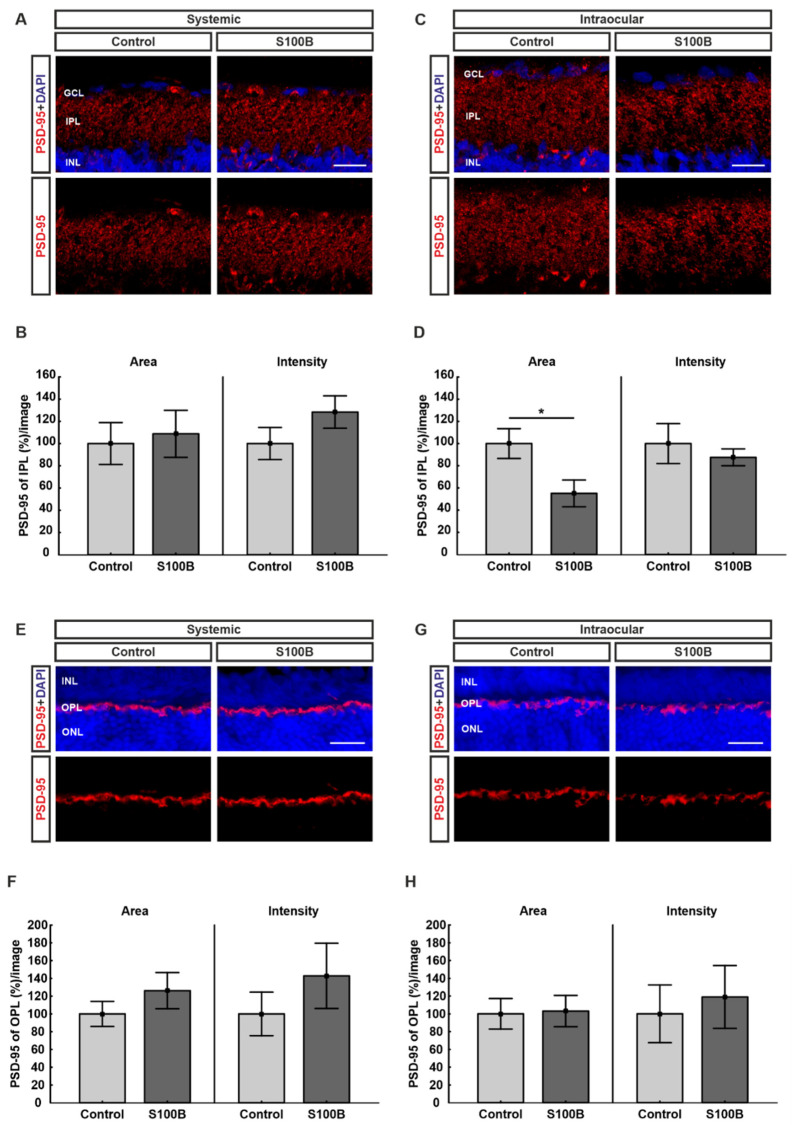
PSD-95 decreased in intraocular-injected animals. (**A**) Retinas from animals with systemic immunization were labeled against PSD-95 (red) in the IPL (*n* = 7/group). DAPI stained the cell nuclei (blue). (**B**) No differences were noted regarding the stained area (*p* = 0.76) as well as the intensity (*p* = 0.19) of the staining in the IPL between both groups. (**C**) Retinas from intraocular-injected animals were stained with PSD-95 (red) in the IPL (*n* = 6/group). Cell nuclei were visualized by DAPI (blue). (**D**) Animals with intraocular S100B injection showed a diminished PSD-95 labeled area (*p* = 0.03) in the IPL in comparison to controls. Regarding the percentage of intensity of the staining, no alterations were observed between both groups (*p* = 0.53). (**E**) Retinas from systemically immunized animals (*n* = 7/group) were stained with PSD-95 (red) in the OPL. Cell nuclei were labeled with DAPI (blue). (**F**) Statistical examinations showed no differences between both groups with systemic immunization regarding the PSD-95-stained area (*p* = 0.31) and the intensity (*p* = 0.35) of the staining in the OPL. (**G**) Retinas from intraocular-injected animals (*n* = 7/group) were labeled against PSD-95 (red) in the OPL. DAPI visualized cell nuclei (blue). (**H**) In animals with intraocular application, no alterations could be observed between both groups regarding the stained area (*p* = 0.90) and the intensity of the staining (*p* = 0.69) in the OPL. Abbreviations: GCL: ganglion cell layer; IPL: inner plexiform layer; INL: inner nuclear layer; OPL: outer plexiform layer; ONL: outer nuclear layer. Values are mean ± SEM. Scale bars: 20 µm. * *p* < 0.05.

To evaluate possible alterations in the excitatory post-synapses, retinas were stained with PSD-95 (Figure 3A,C,E,G). Statistical analyses were performed in two different regions of the retina and showed that PSD-95 remains unchanged in systemically immunized S100B animals relating to the stained area (108.79 ± 21.1%; *p* = 0.76) compared to controls (100.00 ± 18.8%; Figure 3B) in the inner plexiform layer (IPL). Furthermore, no alteration in the intensity of the staining between the S100B (128.33 ± 14.5; *p* = 0.19) and the control group (100.00 ± 14.4%) was observed (Figure 3B) in the IPL. Similar outcomes were found regarding the outer nuclear layer (OPL), where the percentage of labeled area (126.22 ± 20.3%; *p* = 0.31) remained unchanged in comparison to controls (100.00 ± 14.0%) and the intensity of the PSD-95 staining of the S100B (142.80 ± 36.6%; *p* = 0.35) remained unchanged in comparison to the control group (100.00 ± 24.5%; Figure 3F).

For the intraocular injections, the percentage of labeled PSD-95 area in the IPL was diminished in S100B animals (55.08 ± 12.0%; *p* = 0.03) compared to controls (100.00 ± 13.44%; Figure 3C). The intensity of the staining showed no alterations between the S100B (87.57 ± 7.6%; *p* = 0.53) and the control group (100.00 ± 17.9%) in the IPL (Figure 3C). Regarding the OPL, no differences in the PSD-95 labeled area were encountered in S100B animals (103.14 ± 17.5%; *p* = 0.90) compared to controls (100.00 ± 17.1%; Figure 3H). In comparison to controls (100.00 ± 32.4%), the intensity of the PSD-95 staining in the OPL remained unchanged as well (118.95% ± 35.3%; *p* = 0.69; Figure 3H).

### 2.4. Upregulation of NMDA Receptors in Both Models

**Figure 4:**
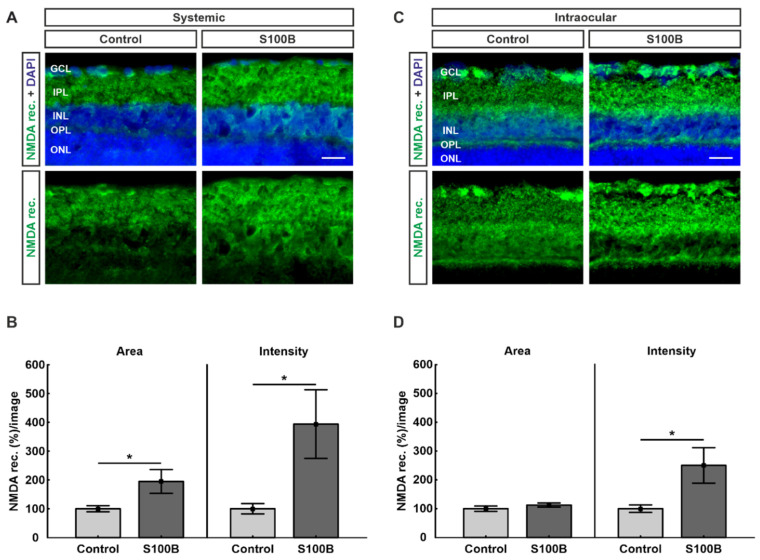
Upregulation of NMDA receptors. (**A**) Retinas from systemically immunized animals (*n* = 7/group) were stained with antibodies against NMDA receptors (green). Cell nuclei were visualized with DAPI (blue). (**B**) In comparison to controls, the NMDA receptor signal area (*p* = 0.04) as well as the intensity (*p* = 0.03) of the staining were significantly increased in the S100B group. (**C**) Retinas from the intraocular injection study (*n* = 6–7/group) were labeled against NMDA receptors (green). Cell nuclei were stained with DAPI (blue). (**D**) Statistical analyses showed that there was significantly higher staining intensity in the S100B group in comparison to controls (*p* = 0.03). The percentage of labeled area was very similar in both groups (*p* = 0.29). Abbreviations: GCL: ganglion cell layer; IPL: inner plexiform layer; INL: inner nuclear layer; OPL: outer plexiform layer; ONL: outer nuclear layer; NMDA rec.: NMDA receptor. Scale bars: 20 µm. Values are mean ± SEM. * *p* < 0.05.

Due to the known interaction between PSD-95 and the NMDA receptor 1, retinas were stained with antibodies against NMDA receptor 1 (Figure 4A,C). In comparison to controls (100.00 ± 10.5%), the percentage of labeled area of the NMDA receptors 1 was significantly increased in the systemic S100B group (194.77 ± 41.2%; *p* = 0.04; Figure 4B). Furthermore, a significantly higher intensity of the NMDA receptor 1 staining could be found in the systemically immunized S100B group (393.81 ± 119.3%; *p* = 0.03) compared to the control group (100.00 ± 17.8%; Figure 4B).

While the stained area in the intraocular model showed no alterations between the S100B group (112.66 ± 7.0%; *p* = 0.29) and controls (100.00 ± 9.3%), the intensity of the NMDA receptor 1 staining was significantly increased in the S100B group (250.27 ± 61.5%; *p* = 0.03) in comparison to controls (100.00 ± 12.9%; Figure 4D).

### 2.5. Excitatory Pre-Synapses Were Remodeled in Intraocular-Injected Animals

**Figure 5:**
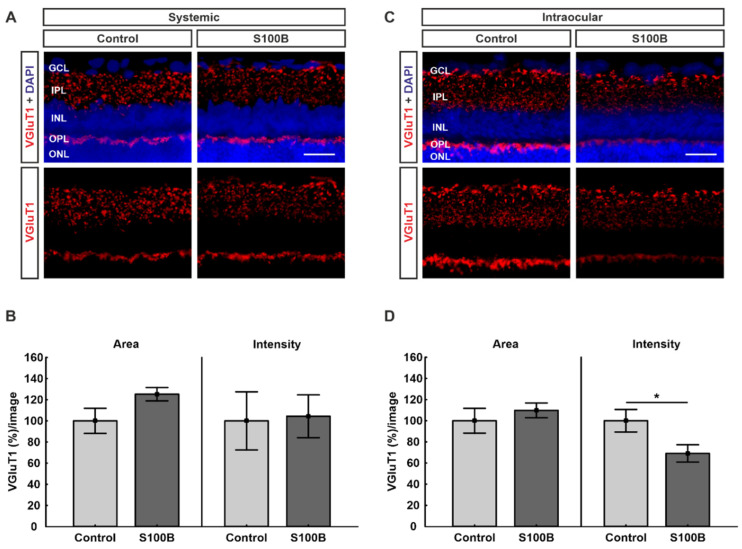
Remodeled VGluT1 in intraocular-injected animals. (**A**) Retinas from animals with systemic immunization (*n* = 7/group) were labeled against VGluT1 (red). Cell nuclei were labeled with DAPI (blue). (**B**) The statistical evaluation of the VGLUT1-stained area showed no significant difference between both groups, though there was a trend towards greater signal area in the S100B group (*p* = 0.08). The intensity of the staining remained unchanged (*p* = 0.90). (**C**) Retinas from animals with intraocular application (*n* = 7/group) were labeled against VGluT1 (red). DAPI visualized cell nuclei (blue). (**D**) The evaluation of the percentage of labeled area revealed no alterations between both groups (*p* = 0.48). In comparison to controls, the intensity of the staining in the S100B group was significantly lower (*p* = 0.04). Abbreviations: GCL: ganglion cell layer; IPL: inner plexiform layer; INL: inner nuclear layer; OPL: outer plexiform layer; ONL: outer nuclear layer. Scale bar: 20 µm. Values are mean ± SEM. * *p* < 0.05.

Excitatory pre-synapses were represented by staining against the vesicular glutamate transporter 1 (VGluT1; Figure 5A,C). The statistical evaluation of the VGluT1-stained area showed no significant difference in systemically immunized animals, though there was a trend towards greater signal area in the S100B group (125.22 ± 6.2%; *p* = 0.08) compared to controls (100.00 ± 11.9%; Figure 5B). Regarding the intensity, no alterations could be detected between the S100B animals (104.31 ± 20.2%; *p* = 0.90) and the control group (100.00 ± 27.5%; Figure 5B).

Animals with intraocular injection of S100B showed no alterations of the VGluT1-stained area (109.81 ± 6.9%; *p* = 0.48) in comparison to controls (100.00 ± 11.7%; Figure 5D). Interestingly, there was a significantly lower intensity of anti-VGluT1 staining in the intraocular-injected S100B group (69.12 ± 8.1%; *p* = 0.04) compared to the control animals (100.00 ± 10.7%; Figure 5D).

## 3. Discussion

While glaucoma is one of the most common causes of blindness worldwide, its exact pathomechanism still remains unclear. Recent studies have suggested that synapse loss mediated through the immune system could be involved in glaucoma genesis [35,36]. In this current study, we used two different S100B-based glaucoma-like models to determine synaptic alterations before and during neuronal degeneration. In the retina, chemical synapses are formed between amacrine and interplexiform cells and between photoreceptor and bipolar cells (the latter called ribbon synapses) [37].

In EAG rats, which received a systemic S100B immunization in combination with HSP27, a significant loss of RGCs, bipolar cells, and amacrine cells was accompanied by a significantly lower synapse signal after 28 days. Besides less post-synapses visualized by PSD-95, there were also fewer pre-synapses labeled with the pre-synaptic active zone protein bassoon [30]. PSD-95 is a scaffolding protein located at the postsynaptic density [38] and is known for regulating activity-dependent synaptic plasticity and its transmission [39,40]. Not only in this EAG model but also in a model of ocular hypertension (OHT) glaucoma in mice, a reduction of excitatory synapses visualized by PSD-95 was observed prior to structural changes but associated with functional changes [41]. In another OHT model in mice, the loss of RGCs and ribbon synapses was accompanied by a significant enhancement and subsequent, slightly but not significant, reduction of PSD-95 and synaptophysin, a pre-synaptic vesicle protein [42]. In our current study, we noted no alterations regarding the PSD-95 staining in animals systemically immunized with S100B. However, animals that received S100B intraocularly showed a significant reduction of PSD-95 area after 14 days. As RGC degeneration in the intraocularly injected animals occurs earlier than in the systemically immunized ones [27,28,29], we assume that synapse alterations have a similar response. We hypothesize that excitatory post-synapses will degenerate at a later point in time in the systemic S100B model as well.

It has been well investigated that PSD-95 interacts with the NMDA receptor 1 [31,32,33] and stabilizes and increases it as well as its channel opening rate [43,44]. NMDA receptors are subtypes from ionotropic glutamate receptors and in the rat retina are expressed throughout the outer and inner plexiform, inner nuclear, and ganglion cell layer [45]. NMDA receptors are in general composed of different subunits, including GluN1, GluN2 (GluN2A–GluN2D), and GluN3 (GluN3A, GluN3B) [46]. In our study, a significantly higher NMDA receptor 1 (GluN1) signal was detected in systemic as well as in intraocular S100B rats after 14 days. In mice deficient in different GluN2 subunits, it was shown that a lack of GluN2B and GluN2D protected RGCs from cell death through NMDA-induced excitotoxicity [47,48]. Since excitotoxicity through glutamate [49,50] and its analogue NMDA [51,52] is a major concern in retinal and optic nerve damage as well as other neurodegenerative diseases [53,54], we assume that an upregulation of the NMDA receptor 1 could be an indication for excitotoxicity taking place in both animal models in this study. In patients affected with the neurodegenerative Huntington’s disease, a high expression of NMDA receptors led to an early neuronal loss [55]. Therefore, we additionally assume that an upregulation of NDMA receptors could cause the interacting post-synapses to subsequently degenerate at a later point in time in both models. In a rat OHT model, heightened NMDA receptor contribution to synaptic transmission was observed in the superior colliculus after RGC degeneration [56]. Thus, a different suggestion is that the upregulation of NMDA receptors in our IOP-independent models could be a counterregulatory step to support synaptic function prior to and simultaneously with RGC death.

To investigate inhibitory post-synapses in both models, we marked retinas against gephyrin. By binding glycine and GABA-A receptors to the post-synaptic cytoskeleton, gephyrin functions as an anchor protein [57,58]. Gephyrin is known to interact with several signaling molecules, and its role as a scaffolding protein is regulated through different post-transcriptional and post-translational alterations and various forms of mRNA splicing [57]. In EAG mice that received a systemic immunization with optic nerve antigen homogenate (ONA), a significantly lower inhibitory post-synapses signal was visualized by anti-gephyrin in a later stage of degeneration, after six weeks. Gephyrin is encoded by the GPHN gene, and a significant downregulation of the *Gphn* mRNA was also found [59]. In our study, neither the systemically immunized animals nor the intraocular-injected ones showed any differences regarding the synapses labeled by gephyrin after 14 days. Probably, 14 days is too early in the degeneration process in both models and the inhibitory post-synapses could be affected by degeneration at a later point in time.

Earlier studies showed an interaction between gephyrin and different subtypes of GABA-A receptors [34,57,60]. GABA-A receptors are fundamental for the inhibitory neurotransmission in the central nervous system [61], but under certain circumstances they can function in an excitatory way [62]. In the rat retina, GABA-A receptors with the subunit α3 are expressed throughout the inner plexiform, the inner nuclear, and the ganglion cell layer [63]. A previous study revealed that postsynaptic GABA-A receptors could support the development of functional inhibitory synapses in embryonic rat neurons [64]. In a rat OHT model as well as in DBA/2J mice, RGC loss was associated with increased expressions of GABA-A/B receptors in the arcuate nucleus [65]. In both of our S100B-based glaucoma-like models, a significantly higher GABA-A receptor α3 signal was found. It is possible that the upregulation of GABA-A receptors is the first counterregulatory step to support synaptic function, followed by degeneration of the interacting inhibitory post-synapses later on. Another suggestion is that GABA-A receptors do not behave protectively, but rather intensify retinal damage, since it was noted that a GABA-A receptor agonist induced RGC death in a model of oxidative stress [66].

Representing excitatory pre-synapses, VGluT1 stabilizes and fills the synaptic vesicles with the excitatory neurotransmitter glutamate [67]. In the retina, glutamate transporter proteins are the only effective way to remove glutamate from the extracellular fluid [68]. In a normal-tension glaucoma animal model, RGC death and optic nerve degeneration was observed in mice that were lacking the glutamate transporters GLAST or EAAC1, suggesting that glutamate transporters are necessary to prevent excitotoxicity [69]. Since VGluT1 is encoded by the *Slc17a7* gene, a significantly lower level of its mRNA expression was found in ONA-immunized EAG mice after six weeks [59]. In the current study, we noted a tendency towards a higher VGluT1 signal in the systemically immunized S100B animals and a significant downregulation in rats receiving S100B intraocularly after 14 days. Hence, we suppose that in the EAG model an early upregulation of excitatory pre-synapses could be the first counterregulatory step, followed by degeneration at a later point in time.

Besides glaucoma, synaptic degeneration seems to be a common early characteristic throughout many neurodegenerative diseases. In an animal model of age-related macular degeneration, the expression of the synapse-associated proteins synaptophysin and VGluT1 was significantly diminished [70]. Also in retinitis pigmentosa, alterations in synapse and glutamate receptors are supposed to take part in its pathomechanism [71,72,73]. In addition to synapse alterations occurring in ocular diseases, they also play a decisive role in other diseases, especially in relation to Alzheimer’s disease [74,75,76,77], as reduced levels of VGluT1 heighten beta amyloid-induced neuroinflammation and affect synaptic plasticity [78]. Likewise, in an animal model that examined the impact of apolipoprotein E4, lower levels of VGluT1 were noted in retinas [79]. In addition, the diagnosis of Alzheimer’s disease is associated with an abnormal clustering of gephyrin [80] and its lowering correlates with an increasing severity [81]. Gephyrin dysfunctions are further found in other neurological diseases, including stiff-man syndrome, schizophrenia, autism, epilepsy, hyperekplexia, and molybdenum cofactor deficiency [82,83,84,85]. Moreover, schizophrenia and autism are associated with PSD-95 gene mutations [86,87,88].

All these synapse alterations taking place in different neurodegenerative diseases underline the importance of gaining insight into the role of synapse changes relating to glaucoma genesis, as this could possibly be a therapeutic approach. In a glaucoma animal model induced by chronic IOP, intraocular injection of the brain-derived neurotrophic factor increased the number of ribbon synapses [89]. Likewise, therapeutic approaches altering retinal NMDA [90,91] and GABA receptors [65,92] as well as the interaction between them and their synaptic proteins [93] seem to be promising.

A limitation of our study is that synapses were investigated by their quantity of synapse-associated proteins rather than by their actual function. Though in DBA/2J mice no functional impairments could be observed in cells with normal morphology [94], rats with intraocular S100B injection showed a reduction in their electroretinogram amplitudes regarding the a- and b-wave after 14 days [29]. Although no electroretinogram data for systemic S100B immunization are available, rats that received a systemic ONA immunization showed a reduction in their a- and b-wave after 6 weeks [95], and S100B is a component of ONA [96]. The a-wave amplitude represents the electrical activity of the photoreceptors, and the b-wave amplitude reflects the electrical output of the inner nuclear layer. It might well be that some functional alterations happen even prior to morphological alterations in our models. Furthermore, western blot analyses would be a beneficial addition to future studies in order to analyze the quantity of the synapse-associated proteins in another way.

Hence, further investigations are necessary to conclude whether synaptic or receptor-associated alterations are relevant previous events followed by RGC loss in these studied S100B-based glaucoma-like models.

## 4. Materials and Methods

### 4.1. Animals

The studies included six- to eight-week-old male Lewis or Wistar rats (Charles River). All experiments involving animals were carried out with due regard to the Association for Research in Vision and Ophthalmology Statement for the Use of Animals in Ophthalmic and Vision Research. In addition, the studies were authorized by the Animal Care Committee of North Rhine-Westphalia in Germany (approval codes: 84-02.04.2013.A291 and 84-02.04.2013.A442). During the whole experiments, the animals were kept at constant temperature and a 12:12 h circadian rhythm. They received access to food and water ad libitum.

### 4.2. Systemic Immunization

Lewis rats received an intraperitoneal S100B immunization with 200 µg S100B protein (Sigma-Aldrich, St. Louis, MO, USA). Controls were injected with 200 µL of 0.9% sodium chloride solution. Additionally, both groups received 3 µg pertussis toxin and 200 µL Freud’s adjuvant (*n* = 7/group) [27]. Animals were examined using a neurological score system, which pays special attention to the eyes, coordination, behavior, movement, orientation, and signs of paralysis. They were examined daily for one week and then twice a week after immunization. In this study, weight loss and neurological signs were considered as early endpoints.

### 4.3. Intraocular Injection

Wistar rats received an intraocular S100B injection (*n* = 6–7/group). Before intraocular treatment, animals were anesthetized with a mixture of ketamine (50 mg/mL, Ratiopharm, Ulm, Germany) and xylazine (2%, Bayer Health Care, Leverkusen, Germany) followed by a topical anesthetic (Conjuncain, 4 mg/mL, Bausch & Lomb, Rochester, NY, USA) and a mydriatic to dilate the pupil (Tropicamide, 5 mg/mL, Pharma Stulln, Stulln, Germany). In the S100B group, 0.4 µg/µL S100B solution (Sigma-Aldrich) was injected in the vitreous of one eye with a 32-gauge needle (Hamilton, VWR, Radnor, PA, USA) under a stereomicroscope (Zeiss, Jena, Germany) [29]. Controls received 2 µL phosphate-buffered saline (PBS; Biochrome), which also functioned as a solvent for S100B. After the injection, the eyes were treated with an antibiotic ointment (Floxal, Bausch & Lomb). Animals were examined 2 h after injection and on the next day. Once a week, the general behavior and look of the animals was examined Eye bleeding or cataracts were considered as early endpoints in this study.

### 4.4. Tissue Preparation for Retinal Cross-Sections

After 14 days, eyes were enucleated and fixed with 4% paraformaldehyde for 1 h. Afterwards, they were treated with 30% sucrose. Subsequently, they were embedded in Tissue Tek (Thermo Fisher Scientific, Waltham, MA, USA). Retinal cross-sections (10 µm) were cut with a microtome (Thermo Fisher Scientific) and mounted on microscope slides (Histobond, Paul Marienfeld GmbH & Co. KG, Lauda-Königshofen, Germany). The slides were fixed in ice-cold acetone for 10 min.

### 4.5. Immunofluorescence Stainings of the Retinas

To determine diverse synapse and receptor types of the retina, specific antibodies were used (Table 1). Retinal cross-sections were blocked with a solution containing 20% normal donkey serum and 0.1–0.2% TritonX-100 (Sigma-Aldrich) dissolved in PBS (Santa Cruz Biotechnology, Dallas, TX, USA). Next, primary antibodies were diluted in the same mixture, and sections were incubated at room temperature overnight. The incubation with the corresponding secondary antibody was performed for 1 h on the next day. 4′,6 diamidino-2-phenylindole (DAPI; Serva Electrophoresis, Heidelberg, Germany) helped with visualizing the cell nuclei. For each staining, a negative control was performed by using the secondary antibody only [97,98]. In order to provide equal conditions for comparable outcomes, for each staining, antibodies with the same batch numbers were used and the same staining processes were executed. Controls as well as S100B slices were stained at the same time.

### 4.6. Histological Investigation

**Figure A1:**
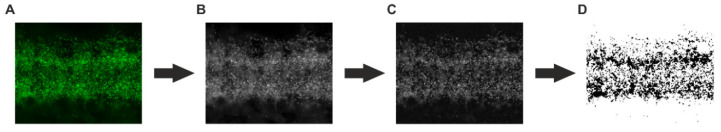
Image processing by ImageJ software using the example of an anti-gephyrin-stained retina. (**A**) Raw image files were added to the ImageJ software. (**B**) Pictures were converted into 32-bit greyscale. (**C**) A previously defined appropriate background was subtracted. (**D**) Then, images were converted into black and white and an upper and lower threshold was set. Suitable thresholds were defined when the black-and-white picture matched the original one.

Two peripheral and two central images were taken per retinal section by using a fluorescence microscope (Axio Imager M1, Zeiss) [99]. Subsequently, an equal area for each staining was adjusted and cut out with the Corel Paint Shop Pro software (V13, Corel Corporation, Ottawa, ON, Canada). To measure the area as well as the intensity of PSD-95, gephyrin, VGluT1, NMDA receptor 1, and GABA-A receptor α3, the ImageJ software (National Institute of Health, Bethesda, MD, USA) was used [100,101] (Figure A1). Raw image files were added to the ImageJ software (Figure A1A). Then, they were converted into grayscale (32-bit; Figure A1B), and a particular rolling ball radius (Table 2) was subtracted to minimize interferences with the background labeling (Figure A1C). Afterwards, images were converted into black and white (Figure A1D), and a proper lower threshold was set for each staining by calculating the mean value of the lower threshold from each image per staining. The upper threshold was set at the highest numeric value out of all of the pictures. Between these defined upper and lower thresholds (Table 2), the percentage of the labeled area as well as the intensity of the staining was measured. To make sure equal conditions for comparable outcomes were existing, for each staining, the same microscope with the same settings and exposure time was used. Furthermore, equal background subtraction and upper and lower thresholds were used for each staining (Table 2).

### 4.7. Statistics

Statistical analyses were carried out by using the Statistica software (V13.3, Dell, Round Rock, TX, USA). The respective S100B groups were compared to their controls by applying Student’s *t*-test. Data are depicted as means ± standard error with *p* < 0.05 considered as statistically significant (* *p* < 0.05; ** *p* < 0.01).

## 5. Conclusions

Two animal models, using systemic or intraocular S100B application, were investigated regarding the contribution of synapse and receptor types alterations. We noted upregulations of the NMDA receptor 1 and the GABA-A receptor α3 at 14 days. While excitatory pre-synapses seem to be upregulated prior to the loss of RGCs, they are downregulated simultaneously with RGC death. Furthermore, excitatory post-synapses and RGCs degenerated at once. However, no quantitative changes regarding inhibitory post-synapses could be detected. These results provide novel insights into the pathomechanism of glaucoma and suggest that changes in NMDA and GABA-A receptors, as well as excitatory pre-synapses, are relevant early events, followed by glaucomatous damage.

## Figures and Tables

**Table 1:** Primary and corresponding secondary antibodies used for immunohistochemistry.

Primary Antibodies			Secondary Antibodies		
Antibody	Company	Dilution	Antibody	Company	Dilution
Anti-GABA-A receptor α3	Synaptic Systems	1:200	Donkey anti-guinea pig Alexa Fluor 488	Jackson ImmunoResearch	1:500
Anti-gephyrin	Synaptic Systems	1:700	Donkey anti-rabbit Alexa Fluor 488	Invitrogen	1:500
Anti-NMDA receptor 1 (GluN1)	Synaptic Systems	1:200	Donkey anti-rabbit Alexa Fluor 488	Invitrogen	1:500
Anti-PSD-95	Millipore	1:300	Donkey anti-mouse Alexa Fluor 555	Invitrogen	1:500
Anti-VGluT1	Synaptic Systems	1:500	Donkey anti-chicken Cy3	Millipore	1:500

**Table 2:** Background subtraction as well as lower and upper thresholds used for staining analysis.

Staining	Background	Lower Threshold	Upper Threshold
GABA receptor	95	1.11	63.11
Gephyrin	50	5.45	79.83
NMDA receptor	80	1.24	61.47
PSD-95 (IPL)	50	3.14	96.69
PSD-95 (OPL)	50	9.09	71.58
VGluT1	50	4.55	81.72
